# Analysis of matrisome expression patterns in murine and human dorsal root ganglia

**DOI:** 10.3389/fnmol.2023.1232447

**Published:** 2023-08-17

**Authors:** Robin Vroman, Rahel S. Hunter, Matthew J. Wood, Olivia C. Davis, Zoë Malfait, Dale S. George, Dongjun Ren, Diana Tavares-Ferreira, Theodore J. Price, Richard J. Miller, Anne-Marie Malfait, Fransiska Malfait, Rachel E. Miller, Delfien Syx

**Affiliations:** ^1^Department of Biomolecular Medicine, Center for Medical Genetics, Ghent University, Ghent, Belgium; ^2^Department of Internal Medicine, Division of Rheumatology, Rush University Medical Center, Chicago, IL, United States; ^3^Department of Neuroscience and Center for Advanced Pain Studies, University of Texas at Dallas, Richardson, TX, United States; ^4^Department of Neurology, Feinberg School of Medicine, Northwestern University, Chicago, IL, United States; ^5^Department of Pharmacology, Feinberg School of Medicine, Northwestern University, Chicago, IL, United States

**Keywords:** matrisome, dorsal root ganglion, nociceptor, cell–cell communication, extracellular matrix

## Abstract

The extracellular matrix (ECM) is a dynamic structure of molecules that can be divided into six different categories and are collectively called the matrisome. The ECM plays pivotal roles in physiological processes in many tissues, including the nervous system. Intriguingly, alterations in ECM molecules/pathways are associated with painful human conditions and murine pain models. Nevertheless, mechanistic insight into the interplay of normal or defective ECM and pain is largely lacking. The goal of this study was to integrate bulk, single-cell, and spatial RNA sequencing (RNAseq) datasets to investigate the expression and cellular origin of matrisome genes in male and female murine and human dorsal root ganglia (DRG). Bulk RNAseq showed that about 65% of all matrisome genes were expressed in both murine and human DRG, with proportionally more core matrisome genes (glycoproteins, collagens, and proteoglycans) expressed compared to matrisome-associated genes (ECM-affiliated genes, ECM regulators, and secreted factors). Single cell RNAseq on male murine DRG revealed the cellular origin of matrisome expression. Core matrisome genes, especially collagens, were expressed by fibroblasts whereas matrisome-associated genes were primarily expressed by neurons. Cell–cell communication network analysis with CellChat software predicted an important role for collagen signaling pathways in connecting vascular cell types and nociceptors in murine tissue, which we confirmed by analysis of spatial transcriptomic data from human DRG. RNAscope *in situ* hybridization and immunohistochemistry demonstrated expression of collagens in fibroblasts surrounding nociceptors in male and female human DRG. Finally, comparing human neuropathic pain samples with non-pain samples also showed differential expression of matrisome genes produced by both fibroblasts and by nociceptors. This study supports the idea that the DRG matrisome may contribute to neuronal signaling in both mouse and human, and that dysregulation of matrisome genes is associated with neuropathic pain.

## 1. Introduction

Chronic pain is a common worldwide problem with inadequate treatment options (Cohen et al., 2021; Yong et al., 2022). Intriguingly, many pathological conditions associated with extracellular matrix (ECM) alterations are associated with the presence of chronic pain (Berk et al., 2012; Marini et al., 2017; Iozzo and Gubbiotti, 2018; Malfait et al., 2020, 2021b). Indeed, pain is often the primary reason patients seek medical attention for complex diseases such as osteoarthritis, and for heritable connective tissue disorders like Ehlers-Danlos Syndromes, Marfan Syndrome, and osteogenesis imperfecta (Dean, 2007; Neogi, 2013; Nghiem et al., 2018; Perrot et al., 2019; Malfait et al., 2021a). Nociception lies at the basis of pain perception. Nociceptors innervating peripheral tissues are activated by a painful stimulus. The generated pain signal gets transduced to dorsal root ganglia (DRG), which are a part of the peripheral nervous system and contain the cell bodies of the sensory neurons (Woolf and Ma, 2007; Haberberger et al., 2019). From the DRG, the pain signal is propagated to the spinal cord and brain, where the signal is consciously perceived as pain. The development of chronic pain involves changes at all levels of the nervous system that modify how an acute transient pain signal is processed and transformed into persistent pain. In the DRG, these pain-associated alterations can include changes in transcription patterns as well as an influx of immune cells (Bangash et al., 2018; Raoof et al., 2018; Miller et al., 2020).

The ECM is a dynamic and interactive three-dimensional network consisting of a large variety of macromolecules that provides structural support and mechanical properties to cells and tissues, including the nervous system (Baeten and Akassoglou, 2011; Brizzi et al., 2012; Kendall and Feghali-Bostwick, 2014; Theocharis et al., 2016; Karamanos et al., 2021). Although the ECM constituents are fundamentally the same, all tissues have a unique ECM composition and topology, adapted to meet their functional requirements (Davis et al., 2019; Teuscher et al., 2019; Tellman et al., 2022). Dysregulation of the ECM organization pathways has been associated with mouse models of chronic pain (Parisien et al., 2019). However, an exact overview of which ECM genes are expressed in DRG tissue and by which cell types is lacking, which prohibits further understanding of the roles the ECM plays in nociceptive functioning. Naba et al. created a list of “matrisome” genes as an ECM framework, which contains structural core matrisome genes, such as glycoproteins, collagens, or proteoglycans, as well as matrisome-associated genes, including signaling molecules and enzymes (Naba et al., 2012; Shao et al., 2019). Therefore, the goal of this study was to integrate bulk, single-cell, and spatial RNA sequencing (RNAseq) datasets with immunohistochemistry (IHC) and RNA *in situ* hybridization data to investigate the expression and cellular origin of matrisome genes in male and female murine and human dorsal root ganglia (DRG). In addition, by using bulk RNAseq data from individuals with or without neuropathic pain, we demonstrate that matrisome genes are differentially expressed in these groups and show potential interactions between cell types expressing these genes in the DRG through ligand-receptor analyses. Together, these studies may lead to identification of novel therapeutic targets for chronic pain.

## 2. Materials and methods

### 2.1. Murine DRG bulk RNA sequencing

Animal experiments were approved by the Ethical Committee of Ghent University (ECD20-62). Mice were housed 2 to 5 per cage with food and water *ad libitum* and kept on 12-h light cycles. Fifteen-week-old male (*n* = 6) and female (*n* = 5) wild-type C57BL/6 mice were euthanized by CO_2_ asphyxiation and bilateral lumbar L3-L5 DRG were collected under RNase free conditions, snap frozen and stored at −80°C. Subsequently, RNA extraction was performed using the RNeasy kit with on column DNase digestion as recommended by the manufacturer (Qiagen). Bulk RNAseq was performed using TruSeq Stranded mRNA library prep followed by 150 bp paired-end sequencing on Illumina’s NovaSeq6000 to obtain 30 million paired-end reads per sample. Reads were aligned against the mouse reference genome (GRCm38) with STAR and counted with StringTie v2.0. Transcripts per million (TPM) values were calculated. To determine the cutoff TPM value above which genes were considered expressed, the average TPM value was calculated for all matrisome genes that were only expressed by one of the 11 samples. This led to a cutoff TPM value of 0.1. A publicly available *in silico* list of murine matrisome genes (*n* = 1,110; v2.0 http://matrisomeproject.mit.edu/other-resources/mouse-matrisome/) was used to filter the bulk RNAseq data. For 12 genes listed in the murine matrisome list, the provided gene name was not found in the bulk RNAseq data. For six genes an alias was found that allowed detection in the bulk RNAseq dataset when replaced in the murine matrisome gene list. For six genes (*Ntn3*, *Itlnb*, *Lgals6*, *Gm5347*, *U06147*, and *Prl2c4*), no alias could be identified and no match in the murine bulk RNAseq data was found.

### 2.2. Human DRG bulk RNA sequencing

**Figure 1:**
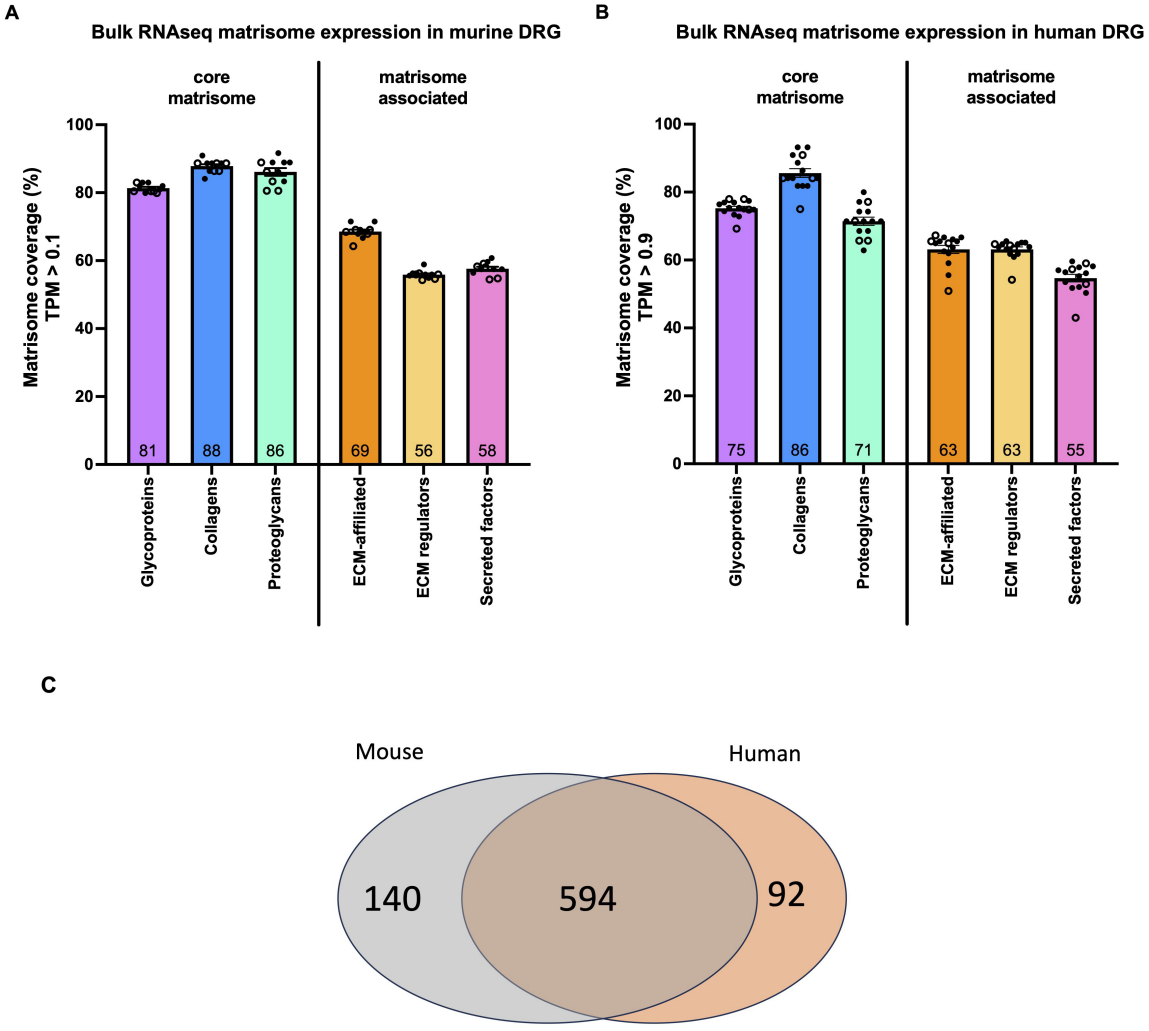
Matrisome gene expression in murine and human DRG. **(A)** Bulk RNAseq was used to identify the percentage of each category of matrisome genes expressed in murine DRG. Dots represent the percentage of expressed genes in DRG collected from one mouse. *n* = 6 male (filled dots), *n* = 5 female (open dots). Total number of genes per category: 194 glycoproteins, 44 collagens, 36 proteoglycans, 165 ECM-affiliated proteins, 304 ECM regulators, and 367 secreted factors. **(B)** Bulk RNAseq was used to identify the percentage of each category of matrisome genes expressed in human DRG. Dots represent the percentage of expressed genes in DRG collected from one individual. *n* = 11 male (filled dots), *n* = 4 female (open dots). Total number of genes per category: 195 glycoproteins, 44 collagens, 35 proteoglycans, 171 ECM-affiliated proteins, 238 ECM regulators, and 344 secreted factors. Number inside the bar represents the mean per group. Mean ± SEM. Human bulk RNAseq data was previously published (Ray et al., 2022). **(C)** Venn diagram showing the overlap between human matrisome genes and their mouse orthologs.

**Figure 2:**
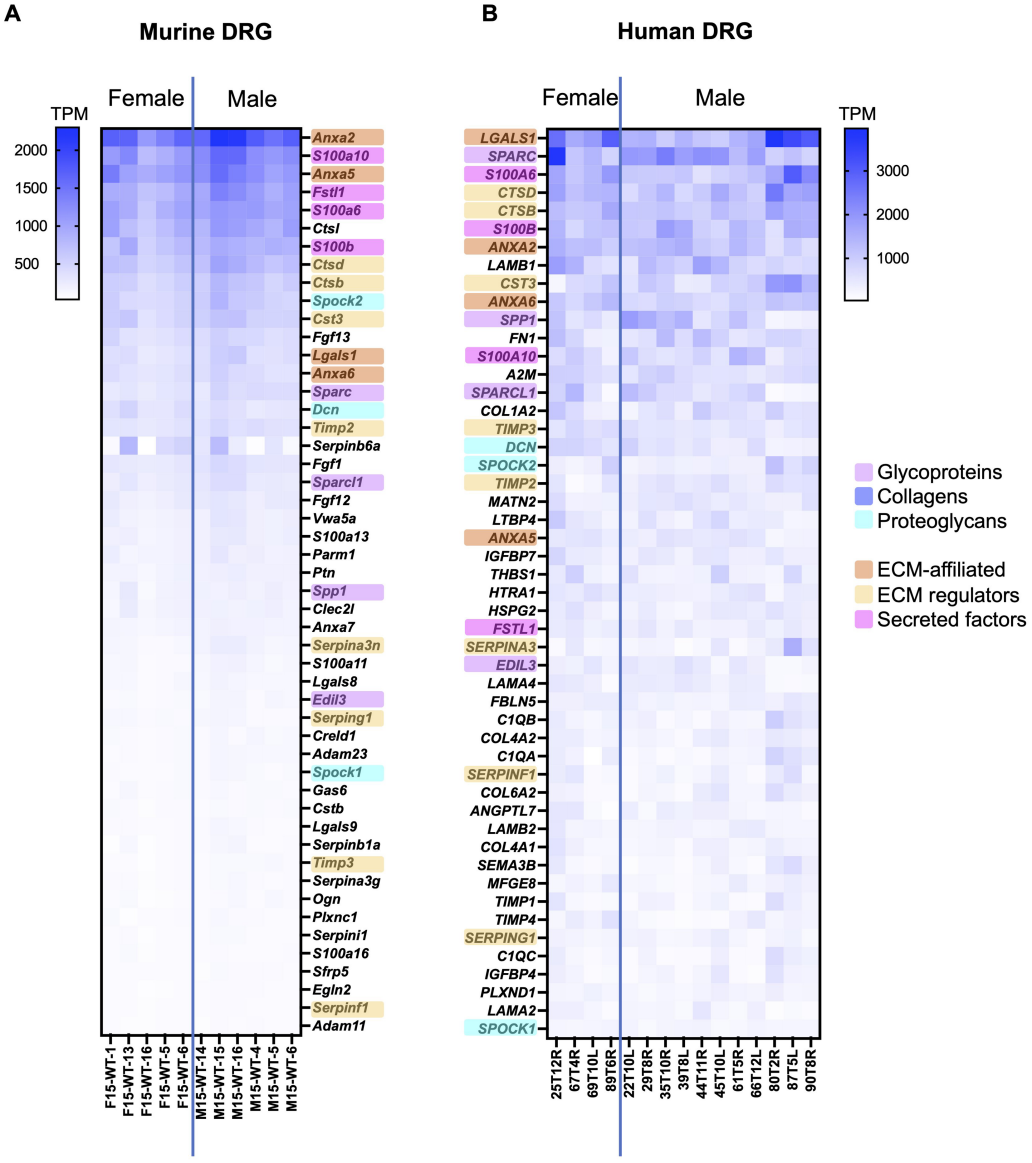
Bulk RNAseq was used to identify the 50 highest expressed matrisome genes in murine and human DRG. **(A)** Murine matrisome genes are ranked by average TPM value across all samples. Male: *n* = 6, female: *n* = 5. **(B)** Human matrisome genes are ranked by average TPM value across all samples. Male: *n* = 11, female: *n* = 4. Orthologues between murine and human datasets have been highlighted in the color corresponding to the matrisome categories. Human bulk RNAseq data was previously published (Ray et al., 2022).

Previously published human DRG bulk RNAseq data was provided by the lab of Dr. Theodore J. Price and can be found on the website^1^ (Ray et al., 2022). Raw RNAseq and processed RNAseq data are available in dbGaP under accession number phs001158.v2.p1. Patients classified with no neuropathic pain (male: *n* = 11, female: *n* = 4, minimum age: 37 years, median age: 61 age, maximum: 79 years) were used for data shown in Figures 1, 2. A predefined *in silico* list of human matrisome genes (*n* = 1,027; v2.0 http://matrisomeproject.mit.edu/other-resources/human-matrisome/) was used to filter the human bulk RNAseq data. TPM values were calculated as described before (Ray et al., 2022) and an average TPM value above 0.9 was considered expressed, by averaging the TPM values of all genes that were expressed in only one of the 15 samples. For 20 genes in the human matrisome list, no match was found for the provided gene name in the human bulk RNAseq dataset. Upon checking the HUGO Gene Nomenclature database an alias or approved gene name could be identified for 14 of these genes and replacing the listed gene name with the alias allowed detection in the bulk RNAseq dataset.^2^ For the six remaining genes (*MUC19*, *MUC2*, *MUC8*, *SERPINA2*, *CCL4L1*, and *MST1L*), no alias could be identified, hence no match could be found in the human bulk RNAseq data. To illustrate the involvement of altered matrisome gene expression in pain states, we analyzed available bulk RNAseq gene lists from human DRG from patients that met the inclusion criteria for neuropathic pain (Ray et al., 2022). We analyzed available gene lists for 4 different conditions: upregulated in male donors classified as pain (MP), upregulated in female pain donors (FP), upregulated in male donors without pain (MN), and upregulated in female donors without pain (FN) and filtered on matrisome genes in the different conditions (Supplementary Table S1).

### 2.3. Murine DRG single cell RNA sequencing

This experiment was approved by the Institutional Animal Care and Use Committees at Rush University Medical Center and Northwestern University. Animals were housed with food and water *ad libitum* and kept on 12-h light cycles. Single cell RNA sequencing (scRNAseq) was performed on pooled L3-L5 DRG cells collected unilaterally from 10, 18-week-old male C57BL/6 mice as described (Obeidat et al., 2023). Cells were dissociated and cell number and viability were analyzed using Nexcelom Cellometer Auto2000 with AOPI fluorescent staining method. Single cell gel beads were generated using 10x Genomics Chromium controller chips at the Northwestern University sequencing core. cDNA and library preparation were performed using 10X Genomics Chromium kits, and samples were sequenced using 50 bp paired-end HiSeq sequencing. Sequencing reads from 9,400 cells were assembled and aligned against the mouse reference genome using the 10x Genomics Cell Ranger v6.0.0. Expression count matrices were analyzed using the Seurat (v4.0.1) R package. Downstream analysis was performed as described before resulting in 8,755 cells for analysis (Obeidat et al., 2023). Cluster names were determined by comparing expression profiles of markers per cluster with mousebrain.org and celltypist.org databases (Supplementary Figures S3A,B; Usoskin et al., 2015; Zeisel et al., 2018; Conde et al., 2022). Raw fastq files and the expression count matrix have been deposited on NCBI GEO (accession number GSE198485).

### 2.4. Intercellular communication analysis

Metadata and data slots of the Seurat object were used to generate a CellChat object using the CellChat R package (CellChat 1.1.3) (Jin et al., 2021). The murine DRG scRNAseq data was preprocessed using CellChat’s standard workflow. CellChat’s database of 2,021 known ligand-receptor interactions in mice was used to infer ligand-receptor interactions and standard pre-processing functions of *identifyOverExpressedGenes* and *identifyOverExpressedInteractions* were applied with CellChat’s default parameters.^3^ Cell–cell communication probability was calculated and communications with fewer than 10 participating cells excluded from analysis. Aggregated cell–cell communication as well as cell–cell communication for the signaling pathways of interest were calculated. Chord, circle, and hierarchy plots were generated using the *netVisual_aggregate()* and *netAnalysis_contribution()* functions.

### 2.5. Human DRG spatial transcriptomics

Previously published human DRG Visium spatial transcriptomics data of 8 donors (Male: *n* = 4, female: *n* = 4) was provided by the lab of Dr. Theodore J. Price and can be found on the website (see text footnote 1) (Tavares-Ferreira et al., 2022). For our in-house sample, one human DRG sample (female; age 96 years, BMI 22.5) was acquired from the Religious Orders Study (ROS) or Rush Memory and Aging Project (MAP) (ROS/MAP) studies as described below (Rush University Medical Center) (Bennett et al., 2018). For both sets of samples, Visium tissue optimization and spatial gene expression protocols were followed as described in the manufacturer’s manual.^4^ Hematoxylin and eosin (H&E) was used as a counterstain for both. Imaging was conducted on an Olympus vs120 slide scanner (Price lab, UTDallas) or ZEISS LSM 980 with Airyscan 2 (University of Illinois at Urbana-Champaign). mRNA library preparation and sequencing were done at the Genome Center in the University of Texas at Dallas Research Core Facilities (Illumina Nextseq 500) as previously published (Tavares-Ferreira et al., 2022), or at Roy J. Carver Biotechnology Center at University of Illinois at Urbana-Champaign (Illumina NovaSeq 6,000) for the in-house sample. The 10x Visium Spatial Tissue Optimization workflow was used to optimize permeabilization conditions for the 2 serial sections of the in-house sample, and the optimal permeabilization time was determined to be 6 min. The data associated with the in-house sample can be found at NCBI GEO (accession number GSE215994).

To assign Visium barcodes to a certain cell type, *DCN* and *SCN10A* were selected as markers to identify VLMC-like (fibroblasts) and nociceptor barcodes, respectively. The 25th quartile was calculated for all of the barcodes having scaled and normalized expression of the gene of interest above 0. Barcodes with expression above this 25th quartile value were considered positive for the gene of interest. These calculations were repeated for selected collagens: *COL1A1*, *COL1A2*, *COL4A1*, COL4A2, *COL6A1*, *COL6A2* and predicted receptors: *CD44*, *SDC1*, *SDC4*, *ITGA1*, *ITGB1*, *ITGA3*, *ITGAV*, and *ITGB8*. Subsequently the sum of double positive barcodes for the gene of interest and *SCN10A* were divided by the total number of *SCN10A* positive barcodes. In addition, the sum of double positive barcodes for the gene of interest and *DCN* were divided by the total number of *DCN* positive barcodes. Double positive barcodes for *SCN10A* and *DCN* were excluded. Finally, we compared the values of both ratios by Wilcoxon matched-pairs signed rank test for each of the 9 samples. The same workflow was used for the matrisome genes differentially regulated in patients with neuropathic pain and their predicted interaction partners by CellChatDB (section 3.5).

### 2.6. Human DRG RNAscope

In house human DRG came from participants in ROS/MAP studies (Bennett et al., 2018). At enrollment, participants agreed to annual clinical evaluation and organ donation at death, including brain, spinal cord, nerve, and muscle. Both studies were approved by an Institutional Review Board at Rush University Medical Center. All participants signed an informed consent, Anatomic Gift Act, and a repository consent to allow their resources to be shared. The DRG were removed postmortem within 12 h and flash frozen as part of the spinal cord removal. Two male human DRG samples (donor 1: age = 82.02 years, BMI = 21.26; donor 2: age = 95.2 years, BMI = 25.17) were acquired from the ROS/MAP studies (Rush University Medical Center). ROS/MAP resources can be requested at https://www.radc.rush.edu.

RNA *in situ* hybridization (ISH) was performed using ACD Bio-Techne RNAscope Multiplex Fluorescent v2 Assay. For human DRG, modifications were made to the protocol to preserve tissue integrity. Briefly summarized, slides were removed from −80°C and immediately submerged in 4% PFA on ice for 40 min. Dehydration was performed using 50%, 75% and two 100% ethanol washes for 5 min each. Hydrogen peroxide (3%) was applied for 10 min. Target retrieval was performed, reducing time in target retrieval buffer to 3 min followed by protease III incubation for 30 min. The remainder of the protocol was performed following manufacturer’s instructions. Probes were used at 1:50 dilution and Opal dyes from Akoya Biosciences were used at 1:100 dilution. Opal dyes 570 (OP-001003) and 650 (OP-001005) were used. *SCN10A* (406291-C3), *COL1A1* (401891-C2), and *DCN* (589521-C3) probes were used. For DAPI staining, Vectashield containing DAPI was used. ACD Bio-Techne positive and negative control probes were conducted prior to start of work. Negative controls were included on every slide. Imaging was performed using an Olympus Fluoview FV10i confocal microscope at 10x and 60x magnification. Multiple planes of focus were captured, but Z-stacks were not produced and instead the optimally focused image was chosen for processing and analysis. Laser intensity was used at ≤9.9% throughout. Images were processed and quantified using Fiji (ImageJ) software (v2.3.0). Only brightness and contrast tools were used to adjust images. In order to quantify cellular expression of *COL1A1*, *SCN10A*, and *DCN*, 10 images at 60x magnification per human DRG were analyzed per sample per staining. First, the total number of cells was identified using both the nuclei staining with DAPI and the phase contrast channels. Each cell was then assessed for expression of each probe, and labeled as single expression, double expression, or no expression. Positive signal was determined when 2 or more positive “dots” per cell were found. The result of the 10 images was averaged per sample and plotted. The total number of cells assessed is indicated in the corresponding figures.

### 2.7. Immunohistochemistry on human DRG

All human tissue procurement procedures were approved by the Institutional Review Boards at the University of Texas at Dallas. Human lumbar DRG from one male and one female organ donor with no notable chronic pain conditions (53 and 45 years old, respectively) were collected within 4 h of cross-clamp, frozen on dry ice and stored in a −80°C freezer until use. One L4 DRG from each donor was embedded in OCT and cut on a cryostat into 20 μm sections that were applied directly onto SuperFrost Plus charged slides. Slides were submerged in 4°C 1% formalin for 15 min, then dehydrated in 50%, 70%, and 100% ethanol for 5 min each. The slides were allowed to dry briefly then a boundary was drawn around the sections using a hydrophobic pen (ImmEdge PAP pen, Vector Labs) and placed in a light-protected, humidity-controlled tray. Sections were incubated overnight in mouse anti-GPC3 (ThermoFisher; #MA5-17083; RRID: AB_2538554; 1:100) and rabbit anti-NeuN (Cell Signaling Technology; #24307; RRID: AB_2651140; 1:1000) diluted in 0.1 M phosphate buffer (PB), with 5% normal goat serum and 0.3% Triton-X 100 (PBS-T). Following this, sections were rinsed twice with PB then incubated with species-specific secondary antibodies (goat anti-mouse conjugated to Alexa Fluor 568; ThermoFisher; #A-11004; 1:500 and goat anti-rabbit conjugated to Alexa Fluor 647; ThermoFisher; A-21247; 1:500) and DAPI (Cayman Chemical; #14285; 1:5000) diluted in PBS-T for 2 h. After two final rinses in PB, a cover slip with a small volume of mounting medium (4% n-propyl gallate, 85% glycerol and 10 mM phosphate buffer, pH 7.4) was applied and secured using nail varnish. Sections were scanned using an Olympus FV3000RS confocal microscope with a 40x oil-immersion lens and a 1.5x zoom. During image acquisition, an empty channel was scanned to visualize autofluorescence, including lipofuscin. Control sections were processed and scanned in the same way but were not incubated with primary antibodies or DAPI.

### 2.8. Statistical analyses

All analyses were carried out in Microsoft Excel, Graphpad Prism 9.4.0 (GraphPad Software, San Diego, CA), the Seurat (v4.0.1) R package, or the Cell Chat (v1.1.3) R package. Bulk RNAseq averages and cutoffs were calculated and ranked in Excel and graphs were created with Graphpad Prism. Core matrisome versus matrisome-associated gene comparisons using bulk RNAseq data were analyzed with unpaired two-tailed t-test. Data are expressed as mean ± SEM, with *n* indicating the number of samples. Human spatial transcriptomics data were analyzed in Seurat and co-expression with cellular markers was compared with a Wilcoxon matched-pairs signed rank test. *p* values less than 0.05 were considered significant.

## 3. Results

### 3.1. Similar percentages of matrisome genes are expressed by murine and human DRG

To obtain an overview of the expression of matrisome genes in DRG, bulk RNAseq was performed on murine and human DRG and expressed genes were filtered against publicly available lists of murine (*n* = 1,110) and human (*n* = 1,027) matrisome genes (Naba et al., 2016).

In murine DRG collected from lumbar levels L3-L5, 65 ± 0.35% of the 1,110 murine matrisome genes were expressed (TPM > 0.1; male: *n* = 6, female: *n* = 5). Of the 274 core matrisome genes, 83% ± 0.38% were expressed, which was significantly higher compared to 59 ± 0.42% of the 836 matrisome-associated genes (*p* < 0.0001) (Supplementary Figure S1A; Supplementary Table S2). Specifically, for the core matrisome, 81% ± 0.38% of glycoprotein, 88% ± 0.55% of collagen, and 86% ± 1.1% of proteoglycan genes were expressed; whereas for the matrisome-associated genes 69% ± 0.61% of ECM-affiliated, 56% ± 0.36% of ECM regulators and 58% ± 0.58% of secreted factors genes showed expression (Figure 1A). We did not see any sex specific differences in overall expression levels in murine DRG (Supplementary Figure S1A).

When interrogating previously published bulk RNAseq data from human DRG with no neuropathic pain for human matrisome genes, a similar trend was observed (Ray et al., 2022). Overall, 64% ± 0.87% of the 1,027 human matrisome genes were expressed (TPM > 0.9; male: *n* = 11, female: *n* = 4). Of the 274 core matrisome genes, 76% ± 0.63% were expressed, which was significantly higher than 59% ± 0.97% of the 753 matrisome-associated genes (*p* < 0.0001) (Supplementary Figure S1B; Supplementary Table S3). In more detail, for the core matrisome we found that 75% ± 0.59% of glycoproteins, 86% ± 1.3% of collagens, and 71% ± 1.2% of proteoglycans were expressed (Figure 1B). For the matrisome-associated genes 63% ± 1.2% of ECM-affiliated, 63% ± 0.73% of ECM regulators, and 55% ± 1.1% of secreted factors were expressed above the threshold (Figure 1B). Furthermore, no sex-specific differences were seen in the ratios of matrisome genes being expressed between male and female human DRG (Supplementary Figure S1B).

Next, we compared the expressed genes in order to determine whether or not the same genes were being expressed in mouse and human. Starting with the human matrisome list, we filtered out all genes expressed in the human DRG that have at least one murine ortholog (660 genes) and found that 87% of these genes were also expressed in murine DRG (594 genes) (Figure 1C; Supplementary Table S4).

These analyses demonstrated that a substantial proportion of the *in silico* defined matrisome genes are expressed in both murine and human DRG and in similar levels in both sexes, however there are some differences in the exact genes expressed in each species.

### 3.2. Highest expressed matrisome genes in murine and human DRG

To examine the highest expressed matrisome genes in murine and human DRG, matrisome genes were ranked based on their average TPM values and analyzed either across all matrisome categories or per matrisome category separately (Figure 2; Supplementary Figure S2; Supplementary Tables S2, S3).

Across all matrisome categories, we focused on the 50 genes with the highest TPM value, which corresponds to approximately the highest 5% of all matrisome genes. For murine DRG, 11 out of the 50 highest expressed genes were core matrisome genes, compared to 23 out of 50 for human DRG. Between mouse and human, 23 matrisome genes overlapped in both 50 highest expressed gene lists (46%) (Figure 2). Seven of these 23 overlapping genes were core matrisome genes (4 glycoproteins and 3 proteoglycans), while 16 overlapping genes belonged in the matrisome-associated group (4 ECM-affiliated genes, 8 ECM regulators, and 4 secreted factors). In particular, matrisome-associated genes had the highest expression in both species and included conserved expression of annexins (*Anxa2*, *Anxa5*, and *Anxa6*), S100 calcium binding proteins (*S100a6*, *S100a10*, and *S100b*), and cathepsins (*Ctsb*, *Ctsd*, and *Cts3*) (Figure 2).

Among the non-overlapping genes between species, some key differences in expression levels were observed, which may be related to the overall difference in cellular content between mouse and human (Haberberger et al., 2019). For example, more collagen (*COL1A2*, *COL4A1*, *COL4A2*, and *COL6A2*) and laminin (*LAMA2*, *LAMA4*, *LAMB1*, and *LAMB2*) genes made the 50 highest expressed gene list for human compared to mouse, which may reflect the fact that human DRG have more fibrous content than mouse DRG (Haberberger et al., 2019). In contrast, mice had more fibroblast growth factor (*Fgf1*, *Fgf12*, and *Fgf13*) genes in the 50 highest expressed genes list, which have been shown to be mainly expressed by DRG neurons in other published murine datasets (Zeisel et al., 2018). To look in more detail in each matrisome category, we also compiled lists of the 10 highest expressed genes in each category (Supplementary Figure S2). From these lists we can see again that, while many of these genes are conserved between species, the relative expression levels differ in murine and human DRG – this can be particularly noted in the collagens category.

Examination of the heatmap resulting from ranking the matrisome genes according to expression levels revealed less intersample variability per gene in the mouse dataset compared to the human dataset (Figure 2). The smaller variability observed in the mouse samples compared to the human samples was expected based on a more variable cohort of human samples compared to age-matched inbred mice.

Despite the fact that the murine and human matrisome gene ensembles are not identical, we observed overlap in the overall and category-specific highest expressed matrisome genes between murine and human DRG, suggesting translational relevance of these genes.

### 3.3. Cellular distribution of matrisome gene expression in murine DRG

To pinpoint the cellular origin of matrisome gene expression and to elucidate which DRG-resident cells express these genes, we looked at one of our previously published scRNAseq data sets on murine DRG (L3-L5 unilateral, pooled from 10 male mice, 18 weeks of age) (Obeidat et al., 2023). A total of 8,755 cells were clustered into different cell types. Based on cluster specific markers, eight different cell types were identified, including neuronal cells [nociceptors (NOCI) and large diameter neurons (LDN)], supporting cells [Schwann cells (SCHW) and satellite glial cells (SATG)], vascular cell types [vascular leptomeningeal-like cells (VLMC-like)/ fibroblast-like, vascular endothelial cells (VEC), and vascular smooth muscle cells arterial (VSMCA)], and immune cells (IMM) (Supplementary Figures S3A,B) (Usoskin et al., 2015; Conde et al., 2022). As a validation, gene expression for each matrisome category was checked in the scRNAseq data and percentages of expressed genes were overall consistent with the bulk RNAseq results, indicating that the obtained scRNAseq dataset is representative for subsequent matrisome analysis (Supplementary Figure S3C).

**Figure 3:**
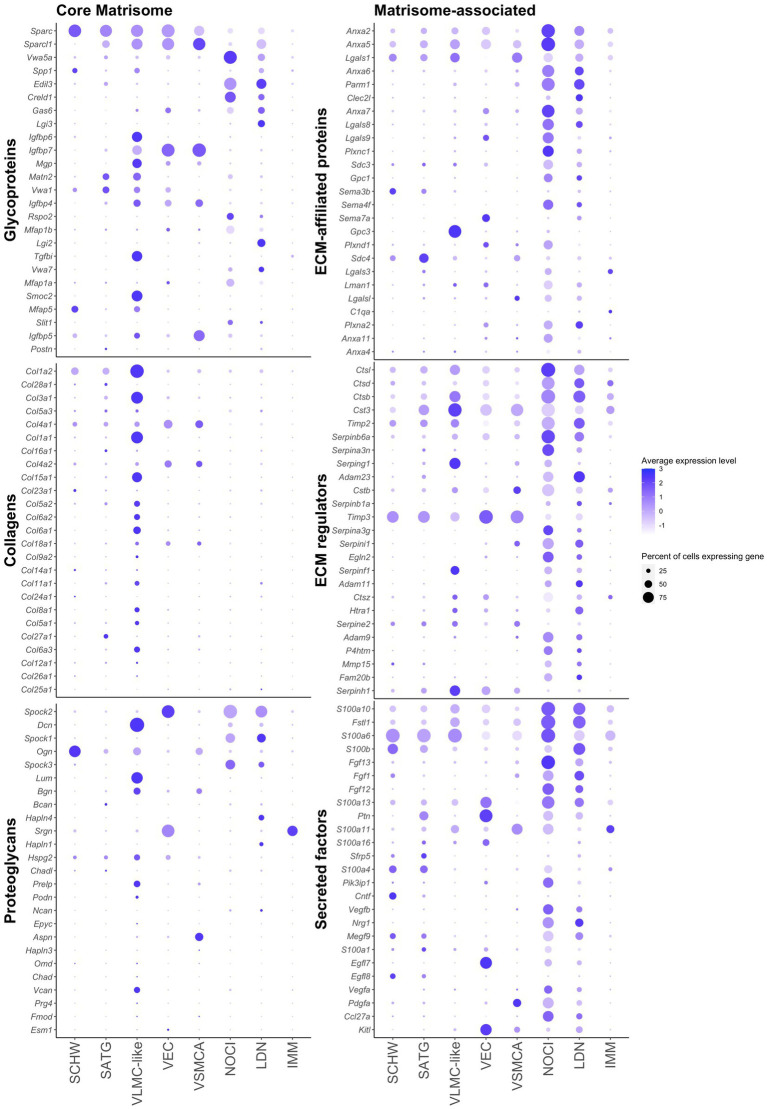
Cellular distribution of the 25 highest expressed genes of each matrisome category in murine DRG determined by scRNAseq. The size of each dot represents the percentage of cells expressing the given gene within a cluster, and the color of each dot corresponds to the average expression (scaled data) across all cells within a cluster for each gene of interest. Schwann cells (SCHW), satellite glial cells (SATG), vascular leptomeningeal-like cells (VLMC-like), vascular endothelial cells (VEC), vascular smooth muscle cells arterial (VSMCA), nociceptors (NOCI), large diameter neurons (LDN), and immune cells (IMM).

To investigate the cellular origin of the matrisome genes, we focused on the 25 highest expressed genes per category obtained from the murine bulk RNAseq data and checked their expression in the different cell clusters from the scRNAseq dataset (Figure 3; Supplementary Table S5). For the core matrisome, cell type-specific expression patterns were observed. Glycoproteins were expressed by all cell types in the DRG, with the exception of immune cells. Although each glycoprotein gene had its own cellular distribution pattern, overall, VLMC-like cells (fibroblasts) were expressing most of the 25 highest expressed glycoproteins. Collagens were predominantly expressed by vascular cell types, more specifically VLMC-like cells (fibroblasts), and not by neuronal or immune cell types. Finally, proteoglycans were primarily expressed by vascular cell types and to a lesser degree by neuronal cells. Two genes deviated from this pattern, *Ogn* and *Srgn*, encoding the proteoglycans encoding osteoglycin and serglycin, respectively. The observed cellular expression profiles of the matrisome genes is consistent with other published datasets such as the mousebrain.org database (Usoskin et al., 2015; Zeisel et al., 2018; Karlsson et al., 2021).

The expression of matrisome-associated genes was predominantly driven by neuronal cell types, with all three categories being expressed by both nociceptors and large diameter neurons (Figure 3). Other DRG-resident cell types also expressed matrisome-associated genes but in general with a lower expression level and by a lower percentage of cells.

### 3.4. Matrisome ligand-receptor interactions in the DRG

To further elucidate the role of the ECM in DRG, interactions between different cell types within the DRG were examined based on the murine scRNAseq data and by using the R package CellChat (v1.1.3). CellChat is an integrated cell–cell communication tool that examines scRNAseq datasets and predicts cell–cell interactions and infers involved pathway networks (Jin et al., 2021).

After analyzing the full set of murine DRG scRNAseq data with CellChat, including both matrisome and non-matrisome genes, we found that all cell types had the potential to interact with each other with various predicted strengths (Supplementary Figures S4A,B). Subsequently, a list of enriched ligand-receptor pathways was generated (Supplementary Figure S4C). Interestingly, the Collagen signaling pathway was the highest ranked and as such contributed the most to the predicted interactions occurring in the DRG.

**Figure 4:**
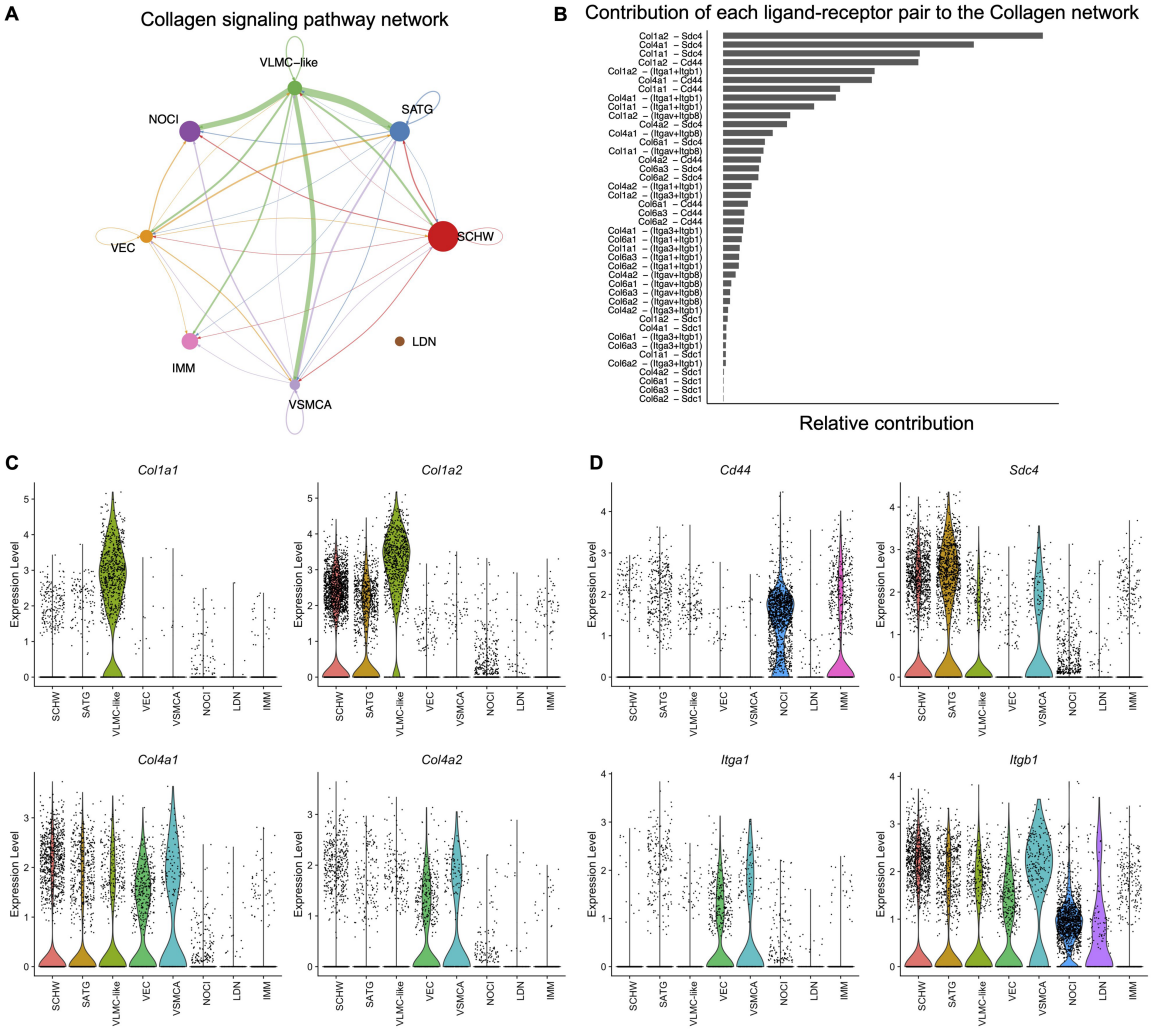
Cell–cell communication in murine DRG inferred from scRNAseq data. **(A)** The inferred Collagen signaling network in murine DRG. Arrow points show the direction of the interaction, and the size of the arrow corresponds with the weight of interaction. Size of the dots corresponds with the relative number of cells per cell cluster. Schwann cells (SCHW), satellite glial cells (SATG), vascular leptomeningeal like cells (VLMC-like), vascular endothelial cells (VEC), vascular smooth muscle cells arterial (VSMCA), nociceptors (NOCI), large diameter neurons (LDN) and immune cells (IMM). **(B)** Each predicted ligand-receptor pair within the Collagen signaling network in murine DRG is ranked based on the relative contribution to the overall Collagen signaling pathway in panel A. **(C)** Violin plots of the most contributing ligand genes in scRNAseq murine DRG: *Col1a1*, *Col1a2*, *Col4a1*, and *Col4a2*. **(D)** Violin plots of the most contributing receptor genes in scRNAseq murine DRG: *Cd44*, *Sdc4*, *Itga1*, and *Itgb1*.

We examined the CellChat predicted signaling pathways of the core matrisome and matrisome associated pathways, starting with the Collagen signaling pathway. The Collagen signaling pathway network showed that VLMC-like cells (fibroblasts) are important for the communication with most of the other cell types in the DRG with the exception of large diameter neurons (Figure 4A). Next, we identified which ligand-receptor pairs contributed the most to the predicted pathway and found *Col1a1*, *Col1a2*, *Col4a1*, and *Col4a2*, encoding types I and IV collagen, as ligands that can interact with the receptors *Cd44* and *Sdc4*, encoding cluster of differentiation 44 and syndecan-4, respectively (Figure 4B). Other receptors contributing to collagen interactions included the integrin pair consisting of *Itga1* and *Itgb1*, which encode the α1 and β1 subunits (Figure 4B). Interactions with less weight involved the type VI collagen encoding genes, *Col6a1*, *Col6a2*, and *Col6a3*, as ligands, and *Sdc1*, encoding syndecan-1, and the integrins *Itga3*, *Itgav*, and *Itgb8*, encoding the α3, αv, and β8 subunits, as receptors (Figure 4B). Examining our scRNAseq data allowed us to examine the cellular sources of these genes in more detail. This revealed that the ligands of the Collagen pathway were primarily expressed by the vascular cell types, in particular the VLMC-like cells (fibroblasts), and to a lesser extent by the supporting Schwann cells and satellite glial cells (Figure 4C; Supplementary Figure S5A). The cell types expressing the interacting receptors were more diverse. *Sdc4* was expressed by satellite glial cells, Schwann cells, VLMC-like (fibroblasts) and VSMCA cells, whereas *Cd44* was expressed by nociceptors and immune cells (Figure 4D). The integrin pair *Itga1* and *Itgb1* was expressed by all other cell types in murine DRG (Figure 4D; Supplementary Figure S5B).

Similar to the Collagen pathway, analysis of the other core matrisome signaling pathways Fibronectin (FN1) and Heparan sulfate proteoglycan (Hspg) (Supplementary Figures S6, S7) showed that the ligand fibronectin was expressed by VLMC-like cells (fibroblasts) and the receptors by the other cell types such as Schwann cells, satellite glial cells, nociceptors, immune cells, and vascular cell types. For the Hspg pathway, the predicted ligand *Hspg2* was expressed by VLMC-like cells (fibroblasts) and vascular endothelial cells, while the receptor, dystroglycan 1 (*Dag1*), was expressed by almost all other cell types, except immune cells.

The matrisome-associated semaphorin 3 (Sema3) pathway was found to originate mainly in Schwann cells, satellite glial cells and nociceptors, while its receptors, neuropilins (*Nrp1 and Nrp2*) and plexins (*Plxna2, Plxna4*, and *Plxnd1*), are expressed by vascular endothelial cells, and neuronal cell types. This pathway demonstrates that Schwann and satellite glial cells are also potentially important for matrisome mediated signaling with neuronal receptors in the DRG (Supplementary Figure S8).

For the other matrisome-associated pathways involving vascular endothelial growth factor (Vegf) or fibroblast growth factor (Fgf) we see a pattern where these ligands are expressed by neuronal cell types (Vegf) or neuronal cell types and Schwann cells (Fgf) and their receptors are expressed by vascular endothelial cells, *Vegfr1* and *Vegfr2* for the Vegf pathway, and by fibroblasts and neuronal cells for *Fgfr1* of the Fgf pathway (Supplementary Figures S9, S10).

**Figure 5:**
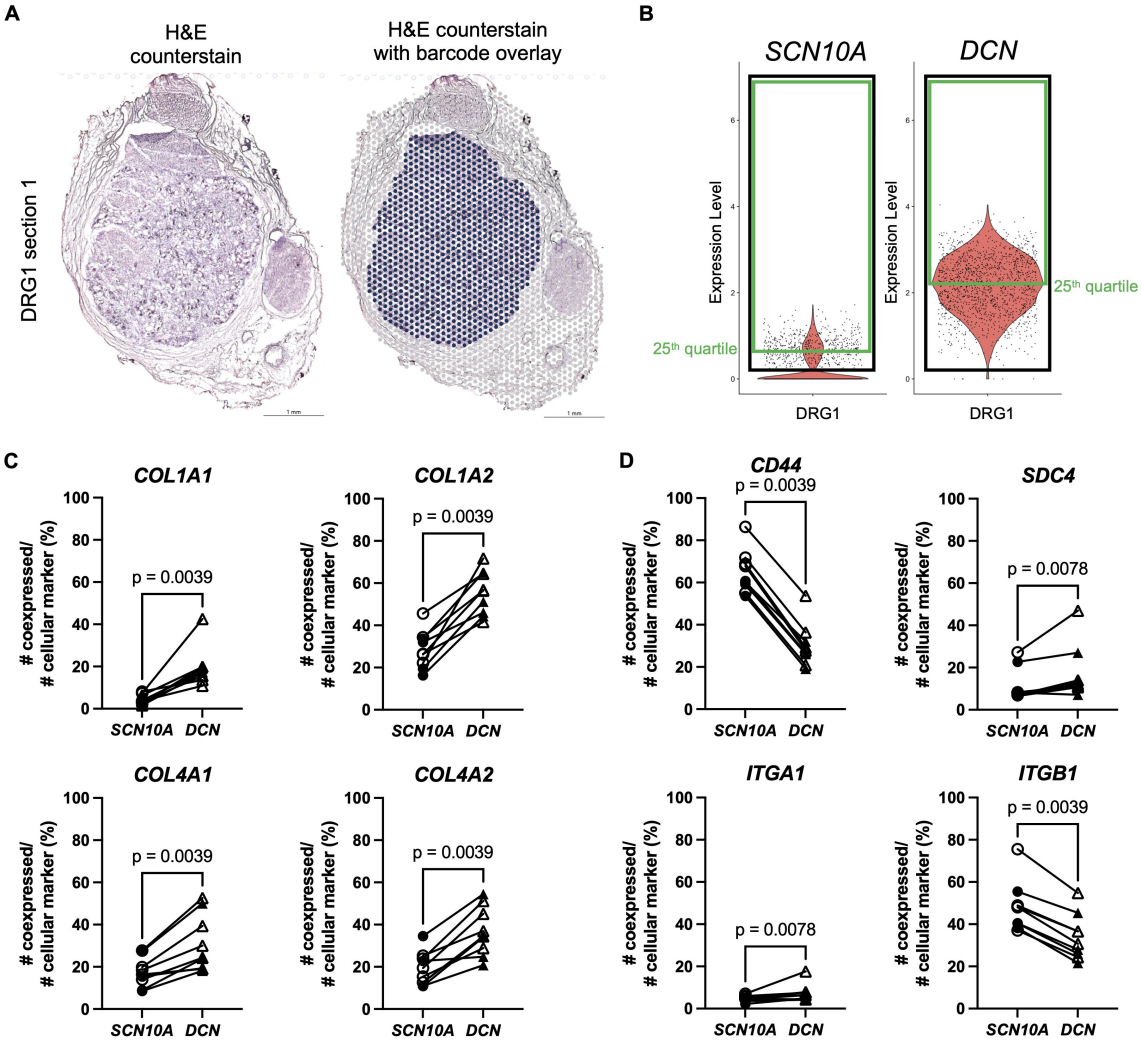
Spatial transcriptomics demonstrates expression of Collagen signaling pathway genes in human DRG. **(A)** Left: H&E counterstain on a section of a human DRG (in-house female DRG sample). Right: section shown with the Visium barcode overlay to demonstrate which barcodes were selected for further analysis in the Seurat R package (in blue). **(B)** After scaling and normalization in Seurat, expression thresholds were established for each gene of interest by using the 25th quartile cutoff (green box) of barcodes >0 (black box) for that gene in each duo of sections per DRG sample. *SCN10A*, and *DCN* are shown here as examples for one DRG, but this method was also applied to genes in panels **(C,D)**. **(C)** Percentage of co-expression of ligands *COL1A1*, *COL1A2*, *COL4A1*, and *COL4A2*, respectively, with *SCN10A* or *DCN*. **(D)** Percentage of co-expression of receptors *CD44*, *SDC4*, *ITGA1*, and *ITGB1*, respectively, with *SCN10A* or *DCN*. *SCN10A*-*DCN* double positive cells were excluded from analyses. Male: *n* = 4 (filled symbol), female: *n* = 5 (open symbol).

This data suggests that matrisome genes are involved in cell–cell interactions in murine DRG and that these interactions involve ligands and receptors expressed by different cell types. The next step was to look if this cell-type specific expression pattern is present in human DRG as well. Therefore, a previously published spatial transcriptomics dataset on human DRG (male: *n* = 4, female: *n* = 4) (Tavares-Ferreira et al., 2022) combined with an unpublished in-house human DRG sample (female: *n* = 1) were examined. To focus on cells within the DRG, barcodes were selected based on the H&E images of 2 serial sections per patient (Figure 5A). We next defined markers for specific cellular subsets in the DRG: *SCN10A* functions as a widely accepted marker for nociceptors, and *DCN* was identified as a marker for VLMC-like cells (fibroblasts) (Shiers et al., 2020). Barcode spots from both sections with expression of a gene of interest above the 25th quartile were considered positive (Figure 5B; Supplementary Figure S11A), after which ratios of co-expression with *SCN10A* and *DCN* were calculated. Based on the murine CellChat data, expression of collagen genes (*COL1A1*, *COL1A2*, *COL4A1*, *COL4A2*, *COL6A1*, *COL6A2*, and *COL6A3*) was evaluated and showed significantly more co-expression with *DCN* compared to *SCN10A* (Figure 5C; Supplementary Figure S11B).

Subsequent examination of their predicted receptors showed significantly more co-expression of *CD44* with *SCN10A* (Figure 5D), consistent with the murine data (Figure 4D), and of *SDC4* with *DCN* (Figure 5D). Integrins showed a variable pattern: *ITGB1*, and *ITGA3* had greater co-expression with *SCN10A*, *ITGA1* and *ITGB8* showed more co-expression with *DCN*, and there was no difference in co-expression for *ITGAV* and *SDC1* (Figure 5D; Supplementary Figure S11C).

Overall, the human spatial transcriptomics data showed consistencies with the murine CellChat data. In particular, the examined collagen genes were preferentially expressed by VLMC-like cells (fibroblasts), and the *CD44* receptor was expressed by nociceptors. Expression of *SDC4* and the examined integrin genes was less clear-cut and is consistent with the finding that these genes were also expressed by other cell types in the murine DRG such as Schwann cells, satellite glial cells, vascular cells, and neuronal cell types.

**Figure 6:**
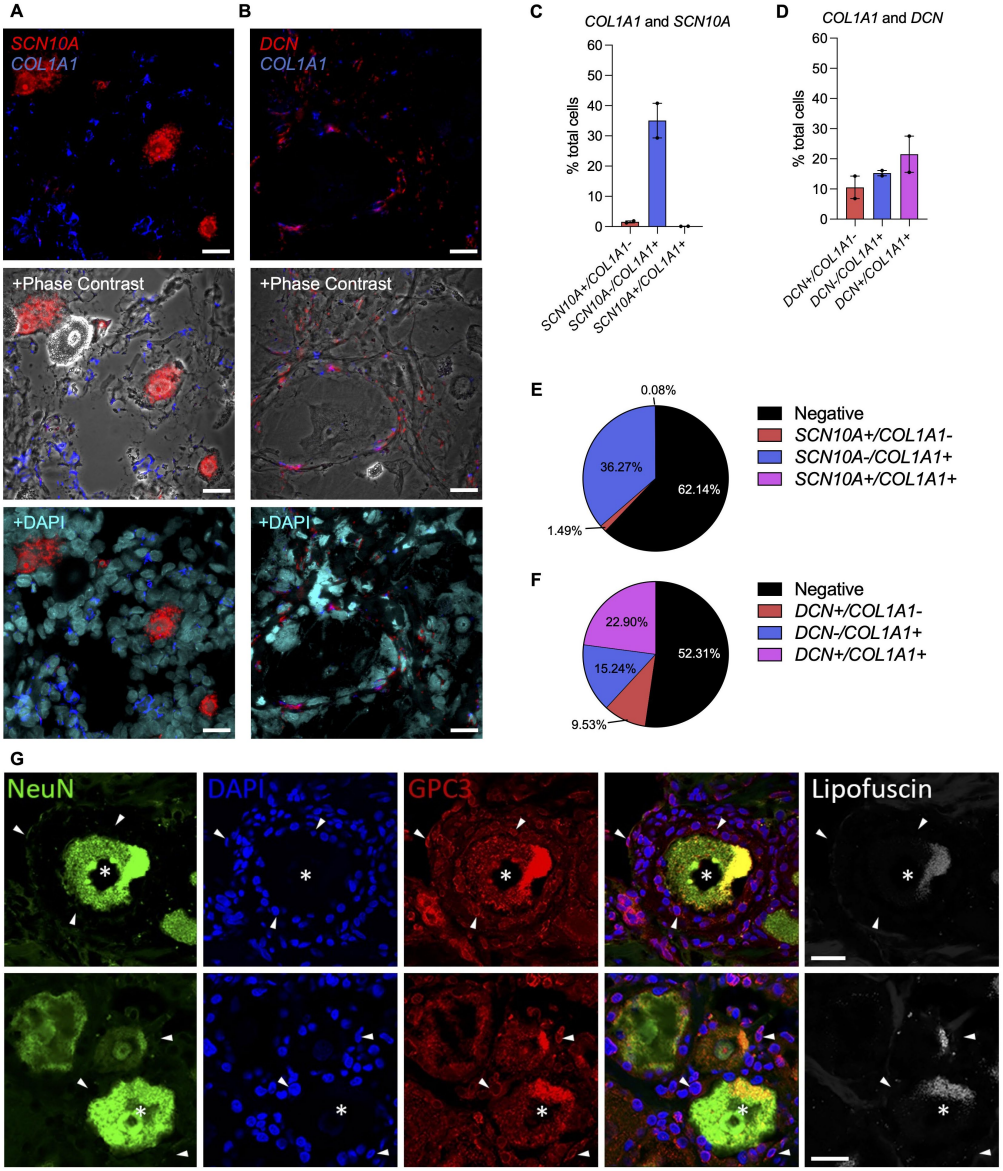
Spatial localization of collagen-producing cells in the human DRG. **(A-F)** Expression of *COL1A1, SCN10A* and *DCN* in human DRG using RNAscope. **(A,C,E)** RNAscope was used to identify and quantify cells expressing *SCN10A* (nociceptor marker) and *COL1A1* or **(B,D,F)** *DCN* (VLMC-like/fibroblast marker) and *COL1A1* in human DRG. Representative sections are shown for *COL1A1* staining with the cellular marker, either with phase contrast or nuclei staining (DAPI) overlay (male donors *n* = 2, number of cells: *SCN10A* = 3,418, *DCN* = 2,768); Mean ± SEM. **(G)** Glypican 3 (GPC3, VLMC-like/fibroblast marker) and NeuN (neuronal marker) expression in human DRG tissue. Empty channel shows lipofuscin autofluorescence as a control. (Top row: male donor *n* = 1, bottom row: female donor *n* = 1). Neurons with GPC3-immunolabelling (asterisks) are surrounded by non-neuronal cells (stained with DAPI), which are often coated with GPC3 immunoreactive protein (examples shown with arrowheads). Scale bar = 25 μm **(A,B,G)**.

The murine scRNAseq data indicated that collagens were minimally expressed by nociceptors. To confirm this in human samples, RNAscope *in situ* hybridization was performed on human DRG sections collected from two donors with probes for *COL1A1* combined with *SCN10A*, a marker for nociceptors, or with *DCN*, a marker for VLMC-like cells (fibroblasts) (Figures 6A–F; Supplementary Figure S12). *SCN10A* positive cells did not show co-expression with *COL1A1*, while in 22% ± 1.8% of DRG cells, co-expression of *COL1A1* with *DCN* was observed. We also found that 15% ± 1.0% of *COL1A1* positive cells did not show co-expression with *DCN*. These findings confirm our earlier results from the murine scRNAseq data in human DRG samples. While it is valuable to know which cell types express certain genes, there can be a different dynamic at the protein level. Hence, we performed IHC on human DRG samples for the VLMC-like cell (fibroblast) cell marker, glypican 3 (GPC3), combined with the neuronal marker NeuN (Figure 6G; Supplementary Figure S13). This demonstrated that VLMC-like cells (fibroblasts) are in close proximity to neuronal cells, increasing the likelihood for the predicted interactions of collagen proteins and their receptors expressed by nociceptors or other cell types in the DRG.

### 3.5. Altered expression of matrisome genes in DRG from patients with neuropathic pain.

To illustrate that matrisome genes can be differentially expressed in pain states, we analyzed an available human DRG pain bulk RNAseq dataset that we used at the start of this manuscript to examine matrisome gene expression in human DRG with no neuropathic pain. We now examined the matrisome gene list to quantify genes that were differentially expressed between pain and no pain conditions (Ray et al., 2022). These DRG were collected from individuals that met inclusion criteria for having neuropathic pain. We looked for matrisome genes in the available gene lists for 4 different conditions: upregulated in male donors classified as having pain (MP), upregulated in female donors with pain (FP), upregulated in male donors without pain (MN), and upregulated in female donors without pain (FN). Across these 4 lists we found 92 matrisome genes that were differentially expressed: 37 in male pain, 29 in female pain, 4 in male no pain, and 22 in female no pain (Supplementary Table S1). Six genes were upregulated in multiple conditions: *ACAN, TGM1*, and *FNDC1* were up in both MP and FP, *S100Z* was upregulated in FP and MN, and *CCL4L2* and *S100A9* were up in MP and FN (Supplementary Table S1; Supplementary Figure S14).

To provide further information regarding the cellular source of these genes, we examined these 92 matrisome genes in the human DRG spatial transcriptomics dataset with no described pain to look at co-expression with the nociceptive marker *SCN10A* and the fibroblast marker *DCN* (Table 1). Twenty-five of these genes showed at least 5% co-expression with one or the other marker (Table 1). Six secreted factors showed greater co-expression with *SCN10A* (Table 1), which is consistent with our murine DRG scRNAseq data, which also showed that nociceptors play a major role in expressing secreted factors (Figure 3). In addition, nineteen genes had more co-expression with *DCN*, (3 glycoproteins, 3 proteoglycans, 2 ECM-affiliated factors, 4 ECM regulators, and 7 secreted factors) (Table 1).

**Table 1:** Human differentially expressed genes were selected based on the matrisome list and the publicly available human DRG bulk RNAseq of patients with no pain or with neuropathic pain (Supplementary Table S1) (Ray et al., 2022).

Division	Category	Gene	Up in	Predicted interaction partners (CellChatDB)
Co-expressed more with *SCN10A*				
Matrisome-associated	Secreted factors	*BDNF*	FP	
Matrisome-associated	Secreted factors	*IL17D*	FP	
Matrisome-associated	Secreted factors	*CXCL13*	MN	*ACKR1, CXCR3, CXCR5*
Matrisome-associated	Secreted factors	*CCL3L3*	MP	*CCR1*
Matrisome-associated	Secreted factors	*CCL3*	MP	*CCR1, CCR5, ACKR2*
Matrisome-associated	Secreted factors	*CCL4L2*	MP, FN	
Co-expressed more with *DCN*				
Matrisome-associated	ECM regulators	*ADAMTSL2*	FN	
Core matrisome	ECM glycoproteins	*IGFBP6*	FN	
Core matrisome	ECM glycoproteins	*MFAP5*	FN	
Matrisome-associated	Secreted factors	*SCUBE1*	FN	
Matrisome-associated	ECM regulators	*SLPI*	FN	
Core matrisome	ECM glycoproteins	*TSKU*	FN	
Matrisome-associated	Secreted factors	*ANGPTL4*	FN	
Matrisome-associated	Secreted factors	*WNT5B*	FN	
Matrisome-associated	ECM-affiliated proteins	*EMCN*	FP	
Matrisome-associated	Secreted factors	*IGF1*	FP	*IGF1R, ITGAV, ITGB3, ITGA6, ITGB4*
Core matrisome	Proteoglycans	*LUM*	FP	
Core matrisome	Proteoglycans	*PRG4*	FP	
Matrisome-associated	ECM regulators	*ADAMTS4*	MP	
Matrisome-associated	Secreted factors	*CCL8*	MP	*CCR1, CCR2, ACKR4, ACKR1, ACKR2*
Matrisome-associated	Secreted factors	*CXCL14*	MP	
Core matrisome	Proteoglycans	*FMOD*	MP	
Matrisome-associated	ECM-affiliated proteins	*SEMA6B*	MP	*PLXNA2, PLXNA4*
Matrisome-associated	ECM regulators	*TGM2*	MP	
Matrisome-associated	Secreted factors	*S100A9*	MP	

Differentially expressed genes were examined in the spatial transcriptomics dataset for co-expression with the nociceptor marker *SCN10A* or the fibroblast marker *DCN*, after which predicted receptors were listed using CellChat. FP, female pain; FN, female no pain; MP, male pain; MN, male no pain.

**Figure 7:**
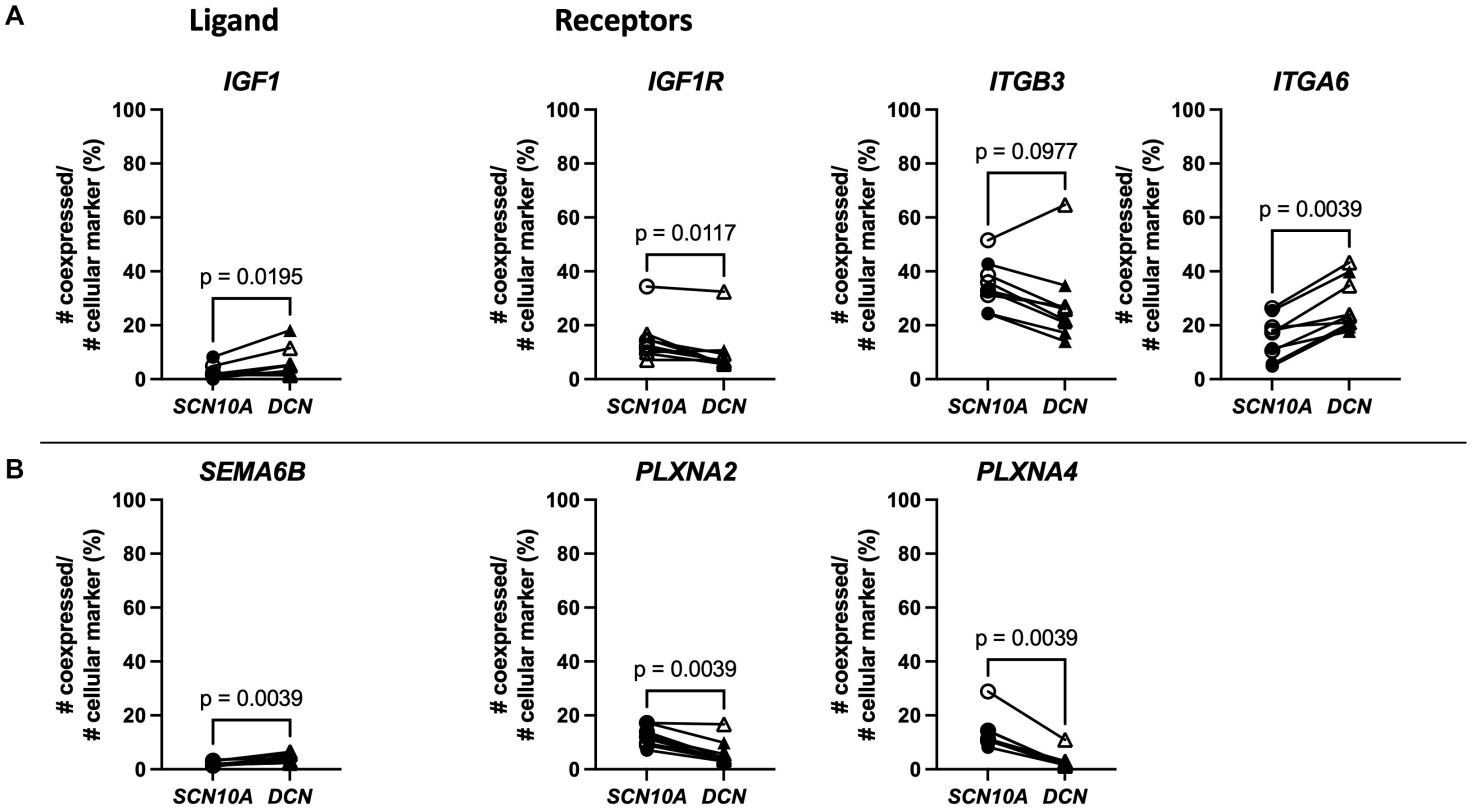
Spatial transcriptomics demonstrates fibroblast expression of two genes upregulated with neuropathic pain (*IGF1* and *SEMA6B*) and nociceptor expression of predicted binding partner genes in human DRG. **(A)** Left: Percentage of co-expression of ligand *IGF1* with *SCN10A* or *DCN*. Right: Percentage of co-expression of receptors *IGF1R, ITGB3, or ITGA6* with *SCN10A* or *DCN*. **(B)** Left: Percentage of co-expression of ligand *SEMA6B* with *SCN10A* or *DCN*. Right: Percentage of co-expression of receptors *PLXNA2, or PLXNA4* with *SCN10A* or *DCN*. *SCN10A*-*DCN* double positive cells were excluded from analyses. Male: *n* = 4 (filled symbol), female: *n* = 5 (open symbol).

A literature search revealed that some of the genes that showed higher co-expression with *DCN* have previously been linked with pain. For example, *IGF1* (Insulin Like Growth Factor 1), was upregulated in FP, and a previous study demonstrated that intraplantar injections of *IGF1* in the hind paw of rats resulted in hyperalgesia, while its antagonist attenuated this effect (Tang et al., 2019). Our data showed that *IGF1* had greater co-expression with *DCN*, while its receptors described in the CellChatDB interaction explorer database *IGFR1* and *ITGB3* had more co-expression with *SCN10A*, and *ITGA6* showed more co-expression with *DCN* (Figure 7A). *SEMA6B* (Semaphorin 6B) was upregulated in MP and recently, semaphorin-plexin signaling has been correlated with chronic pain (Luo et al., 2014; Limoni and Niquille, 2021; Damo and Simonetti, 2022). Here, *SEMA6B* also showed more co-expression with *DCN* (Figure 7B). Interactions of *SEMA6B* with plexin receptor like *PLXNA2* and *PLXNA4* are important in neuronal signaling during development, and here these genes showed more co-expression with *SCN10A*. These examples further suggest that crosstalk between fibroblasts and nociceptors in DRG tissue might correlate with neuropathic pain.

## 4. Discussion

In this study, we investigated matrisome gene expression and its cellular distribution in murine and human DRG. We found that a large percentage of matrisome genes were expressed in both murine and human DRG, with different cell types responsible for producing different types of matrisome components. Single cell and spatial transcriptomics data suggest that the ECM may contribute to cell–cell communications within the DRG, and our RNAscope *in situ* hybridization and IHC data support the idea that fibroblasts (VLMC-like cells) produce collagens and surround the neuronal cell bodies in human DRG. Finally, a subset of matrisome genes associated with either fibroblasts or nociceptors were found to show altered expression in DRG collected from individuals with neuropathic pain. These observations provide a basis for future examination of the functional implications of the matrisome in the DRG both in homeostasis and in chronic pain states.

Our bulk RNAseq transcriptomic experiments showed that a large fraction of matrisome genes are expressed in murine and human DRG. Murine and human matrisome lists are not identical but have a highly similar number of genes per matrisome category. The ratio of core matrisome genes expressed was higher compared to the matrisome-associated genes for murine and human DRG, which is to be expected given the basic structural functions that glycoproteins, collagens, and proteoglycans fulfill. However, the highest expressed genes were matrisome-associated genes such as annexins (*Anxa2*, *Anxa5*, and *Anxa6*), S100 calcium binding proteins (*S100a6*, *S100a10*, and *S100b*) and cathepsins (*Ctsb*, *Ctsd*, and *Cts3*). The annexin family has been studied in DRG for a few decades (Naciff et al., 1996). Annexins are calcium and phospholipid binding proteins and can play a role in ion channel regulation (Gerke and Moss, 2002), including regulation of *Trpv1* (Pei et al., 2011; Zhang et al., 2021) and *Trpa1* (Avenali et al., 2014), ion channels important for nociception. S100 proteins have also been found in the DRG and have been shown to co-localize particularly with large diameter neurons in the DRG (Ichikawa et al., 1997). S100 proteins can modulate neuronal stimulation using their calcium binding properties (Gonzalez et al., 2020), including ion channels in the DRG, by interacting with downstream receptors or mediators of K^+^ channels, Na_V_1.8 channels, or Na^+^ channels (Seo and Svenningsson, 2020). Moreover, S100 proteins have also been shown to attenuate the development of long-lasting pain (Parisien et al., 2022). Cathepsins are lysosomal proteases that are involved in many different processes. Specifically, in a neuronal context, they have been described to be involved in neuronal development and to play a role in neurological diseases of the central nervous system (Yadati et al., 2020). In addition, a role in axon outgrowth of sensory neurons in the DRG has also been described (Tran et al., 2018). Finally, different genes of the *Timp* (tissue inhibitors of metalloproteinase) family were found among the highly expressed genes in both mouse (*Timp2* and *Timp3*) and human DRG (*TIMP1, TIMP2, TIMP3*, and *TIMP4*). Recently, Timp1 has been shown to attenuate pain and hypersensitivity in a murine model of inflammatory pain through both MMP (matrix metalloproteinase) inhibition and receptor-mediated signaling (Knight et al., 2019). These studies show the importance of the highest expressed matrisome-associated genes in our murine and human DRG bulk RNAseq datasets, and demonstrate why it is not surprising that these were among the highest expressed genes given their involvement in neuronal functioning.

Structurally, mouse and human DRG tissue is quite different, with murine DRG showing a higher neuronal density, while human DRG contain thicker layers of ECM throughout (Haberberger et al., 2019). Despite the big advances in the scientific understanding of pain neurobiology in rodents, high quality analgesic treatments for humans remain elusive, highlighting the importance of research on human nervous tissue (Ray et al., 2018; Mogil, 2019; Renthal et al., 2021). Here, we found that in human DRG, higher expression of collagen (*COL1A2*, *COL4A1*, *COL4A2*, and *COL6A2*) and laminin (*LAMA2*, *LAMA4*, *LAMB1*, and *LAMB2*) genes were observed compared to mice. This is consistent with the observation that human DRG have more ECM between neurons, resulting in less dense neuronal content compared to murine DRG (Haberberger et al., 2019). In contrast, mice had higher expression of genes primarily expressed by neurons such as fibroblast growth factors (*Fgf1*, *Fgf12*, and *Fgf13*) (Zeisel et al., 2018).

Recently, crosstalk between neuronal and non-neuronal cells has received increasing attention (Song and Dityatev, 2018; Shinotsuka and Denk, 2022). Gaining precise knowledge about which cells express which molecules is important for future targeted approaches trying to intervene in specific pathways. Our single cell transcriptomics data using murine DRG elucidated the cellular distribution of matrisome gene expression and revealed that while a large percentage of DRG nociceptors and large diameter neurons expressed the matrisome associated genes at high levels, collagens were minimally expressed by neuronal cell types in mice. Knowing which cells express which molecules is the first step, but understanding the interactions between these expressed genes and different cell types is critical for elucidating potential ECM functions in the DRG. Surprisingly, when taking an unbiased approach and inputting our full list of scRNAseq data (including both matrisome genes and non-matrisome genes), we found that the Collagen pathway, a non-neuronal signaling pathway, was the most prominent ligand-receptor pathway represented in the DRG. The main interactions of this pathway involved the pro-α1 and pro-α2 chains of type I procollagen as ligands and CD44 and syndecan-4 as receptors. While the collagens were mainly expressed by VLMC-like cells (fibroblasts), CD44 was mainly expressed by nociceptors and syndecan-4 was expressed by Schwann cells, satellite glial cells, and vascular cell types. Additionally, we also examined these murine DRG interactions in a human spatial transcriptomics dataset. Genes encoding the pro-α1 and pro-α2 chains of type I collagen showed greater co-expression with a VLMC-like (fibroblast) marker, *DCN*, while the *CD44* receptor showed more expression in nociceptors. This suggests how, despite collagens being minimally or not expressed by nociceptors, they could still influence nociceptive signaling.

After injury, the ECM and its associated enzymes have important functions to remove ECM debris from the injury site as well as ECM remodeling to heal the tissue properly. Also, collagens, laminin, or fibronectin are crucial in peripheral nervous tissue regeneration, and enzymes such as tissue type plasminogen activator are also important for remodeling of the ECM network (Kozai et al., 2007; Gonzalez-Perez et al., 2013). Therefore, one can hypothesize that altered ECM homeostasis might have a detrimental effect on nociceptor functioning, leading to neurological conditions such as pain. Indeed, a recent study looked at differential gene expression in DRG taken from both nerve injury and inflammation-induced mouse pain models and identified the “ECM organization” pathway as being the most dysregulated (Parisien et al., 2019). In addition, another study examined differential gene expression in DRG collected from mice in a neuropathic pain model – when we examined this gene list we found that 17/97 of the regulated genes were matrisome genes, and of these matrisome genes, 3 genes overlapped with the differentially expressed matrisome genes in DRG from patients with neuropathic pain we described, *Lum*, lumican, *Prg4*, proteoglycan 4, and *Ccl8*, chemokine (C-C motif) ligand 8 (Bangash et al., 2018). Clinically, there are also examples of collagen-related diseases with unexplained neurological effects such as pain (Malfait et al., 2021a). For example, several heritable connective tissue disorders caused by defects in collagen-encoding or collagen-regulating genes display a pain phenotype, which is phenocopied in their corresponding murine models (e.g., *Col1a1^Jrt/+^* and *Col5a1^+/−^* mice), suggesting that healthy core matrisome and matrisome-associated signaling is crucial for normal neuronal function and pain perception (Abdelaziz et al., 2015; Syx et al., 2020). Receptors that can bind collagen have also been shown to modulate pain. In the literature, CD44 binding with its other ligand, hyaluronic acid, has been shown to have anti-hyperalgesic effects (Ferrari et al., 2018). In contrast, integrin signaling has been implicated in maintaining hyperalgesia in both neuropathic and inflammatory rat models of pain (Dina et al., 2004; Tsuda et al., 2008). In both cases, the role of collagen interactions with these receptors in painful conditions has not been investigated. Taken together, it can be appreciated that the ECM plays an important role in the physiological functioning of the peripheral nervous system.

Interestingly, a recent review suggested the importance of fibroblasts in pain and pointed out that this is an understudied research area (Shinotsuka and Denk, 2022). We propose that our study helps to address this research need. Our data supports the idea fibroblasts and nociceptors are in close proximity in the human DRG and that differential expression of matrisome genes produced by both fibroblasts and by nociceptors is correlated with human neuropathic pain, providing additional evidence that the predicted fibroblast-nociceptor link may have functional relevance. In addition, our findings provide a set of potential targets to pursue this research further.

This study has a few limitations. First, while we have similar numbers per sex for the age matched murine samples, a limitation of this study is that the human bulk RNAseq samples have more variability in age and numbers per group with 11 male and 4 female samples. Therefore, we did not directly assess sex differences. In addition, a more age-matched approach would be preferred but is challenging given the limited availability of human donor samples. Secondly, we only used male mice for our scRNAseq experiment, which inhibited our ability to look for sex-specific differences at this level. Finally, one should note that this study and its calculations are based mainly on transcriptomics data, which does not always correlate to the protein level and the downstream effects of protein activities.

Overall, this work adds to the literature by documenting matrisome gene expression in the DRG, identifying the cellular components producing these molecules, and providing insight on collagens and their interaction partners in mouse and human DRG. This study will serve as a framework for future examination of the functional consequences of these matrisome expression patterns. An interesting avenue for future research would be to test whether targeting ECM-neuronal interactions influences neuronal functions such as pain. Additionally, it will be important to further examine the role of fibroblasts in mediating pain pathways (Shinotsuka and Denk, 2022).

## Data availability statement

The datasets presented in this study can be found in online repositories. The names of the repository/repositories and accession number(s) can be found at: https://www.ncbi.nlm.nih.gov/, GSE198485.

## Ethics statement

The studies involving human participants were reviewed and approved by the Institutional Review Board at Rush University Medical Center and the Institutional Review Boards at the University of Texas at Dallas. The patients/participants provided their written informed consent to participate in this study. The animal study was reviewed and approved by the Ethical Committee of Ghent University and the Institutional Animal Care and Use Committees at Rush University Medical Center and Northwestern University.

## Author contributions

RV, RJM, A-MM, FM, REM, and DS contributed to the conceptualization and design of the study and reviewed and edited the manuscript. RV wrote the first draft of the manuscript. RV, RH, MW, OD, REM, and DS performed data analysis. MW, OD, ZM, DG, DR, DT-F, REM, and DS carried out sequencing or staining experiments. All authors contributed to manuscript revision, read, and approved the submitted version.

## Funding

This work was supported by the Research Foundation Flanders (FWO), Belgium (1842318N and 3G041519 to FM, and 12Q5920N to DS); Ghent University (GOA019-21); Association française des syndromes d’Ehlers-Danlos (AFSED) to FM; The Ehlers-Danlos Society to FM; the Rheumatology Research Foundation (RRF) to A-MM; the National Institutes of Health [National Institute of Arthritis and Musculoskeletal and Skin Diseases (NIAMS)] (grant number R01AR077019 to REM; R01AR060364, R01AR064251, and P30AR079206 to A-MM) and [National Institute of Neurological Disorders and Stroke (NINDS) R01NS111929 to TP]. A-MM was supported by the George W. Stuppy, MD, Chair of Arthritis at Rush University. These funding sources had no role in the study design; in the collection, analysis, and interpretation of data; in the writing of the report; or in the decision to submit the article for publication.

## Conflict of interest

The authors declare that the research was conducted in the absence of any commercial or financial relationships that could be construed as a potential conflict of interest.

## Publisher’s note

All claims expressed in this article are solely those of the authors and do not necessarily represent those of their affiliated organizations, or those of the publisher, the editors and the reviewers. Any product that may be evaluated in this article, or claim that may be made by its manufacturer, is not guaranteed or endorsed by the publisher.

## Figures and Tables

**Table:** 

*Adam*	a disintegrin and metalloproteinase domain
*Anxa*	annexin
*Ccl8*	chemokine (C-C motif) ligand 8
*Cd44*	cluster of differentiation 44
*Col*	collagen
*Cts*	cathepsin
DAPI	4′,6-diamidino-2-phenylindole
*Dag1*	dystroglycan 1
DCN	decorin
DRG	dorsal root ganglia
ECM	extracellular matrix
*Fgf*	fibroblast growth factor
FN	female no pain
*Fn1*	fibronectin 1
FP	female pain
GPC3	Glypican 3
H&E	hematoxylin and eosin
*Hspg*	heparan sulfate proteoglycan
*IGF1*	insulin like growth factor 1
IHC	immunohistochemistry
IMM	immune cells
*Itg*	integrin
*Lam*	laminin
LDN	large diameter neurons
*Lgals*	galectin
*Lum*	lumican
MAP	memory and aging project
MMP	matrix metalloproteinase
MN	male no pain
MP	male pain
*Nrp*	neuropilin
NOCI	nociceptor
*Ogn*	osteoglycin
PFA	paraformaldehyde
*Prg4*	proteoglycan 4
*PLXNA*	plexin receptor like
RNAseq	RNA sequencing
ROS	religious orders study
SATG	satellite glial cell
SCHW	Schwann cell
*SCN10A*	voltage-gated sodium channel alpha subunit 10
scRNAseq	single cell RNA sequencing
*Sdc*	syndecan
SEM	standard error of the mean
*Sema*	semaphorin
*Sfrp*	secreted frizzled related protein
*Srgn*	serglycin
*Timp*	tissue inhibitors of metalloproteinase
TPM	transcripts per million
*Trpa1*	transient receptor potential cation channel, subfamily A, member 1
*Trpv1*	transient receptor potential cation channel, subfamily V, member 1
*VEC*	vascular endothelial cell
*Vegf*	vascular endothelial growth factor
VLMC-like	vascular leptomeningeal-like cells
*VSMCA*	vascular smooth muscle cells, arterial

## Footnotes

^1^ https://paincenter.utdallas.edu/sensoryomics/

^2^ https://www.genenames.org

^3^ http://www.cellchat.org/cellchatdb/

^4^ https://10xgenomics.com/
