# Supplementary material for: Analysis of matrisome expression patterns in murine and human dorsal root ganglia
Source: Front Mol Neurosci. 2023 Aug 17;16:1232447. doi: 10.3389/fnmol.2023.1232447 (PMC10471487; doi:10.3389/fnmol.2023.1232447)
Supplement: Supplementary file 6 [file Data_Sheet_1.docx]

Supplementary Material

Analysis of Matrisome Expression Patterns in Murine and Human Dorsal Root Ganglia

**Robin Vroman, Rahel S. Hunter, Matthew J. Wood, Olivia C. Davis, Zoë Malfait, Dale S. George, Dongjun Ren, Diana Tavares-Ferreira, Theodore J. Price, Richard J. Miller, Anne-Marie Malfait, Fransiska Malfait, Rachel E. Miller*, Delfien Syx***

*** Correspondence:**Co-corresponding Authors: Rachel_Miller@rush.edu; Delfien.Syx@UGent.be

## Supplementary Figures

**Figure S1:** Matrisome expression in murine and human DRG. **(A)** Percentage of expressed genes for core matrisome genes (total 274) vs matrisome associated genes (total 836) calculated from bulk RNAseq of murine DRG. From left to right, male and female plotted together or separately (n = 6 male (filled dots), n = 5 female (open dots), paired *t*-test); **(B)** Percentage of expressed genes for core matrisome genes (total 274) vs matrisome associated genes (total 753) calculated from bulk RNAseq of matrisome genes in human DRG. From left to right, male and female plotted together or separately (n = 11 male (filled dots), n = 4 female (open dots), paired *t*-test). Number inside the bar represents the mean per group. Mean±SEM. Human bulk RNAseq data was previously published (Ray et al., 2022).

**Figure S2:** Overlap of 10 highest expressed matrisome genes between murine and human DRG per matrisome category based on bulk RNAseq. Highest expressed genes are ranked by average TPM value across all samples per matrisome category. (murine n = 6 male, n = 5 female; human n = 11 male, n = 4 female). Human bulk RNAseq data was previously published (Ray et al., 2022).

**Figure S3:** ScRNAseq on murine DRG. **(A)** UMAP projection of scRNAseq of murine DRG, pooled unilateral L3-L5 DRG of 10 male mice at the age of 18 weeks. Different clusters were identified: Schwann cells (SCHW), satellite glial cells (SATG), vascular leptomeningeal-/fibroblast-like cells (VLMC-like), vascular endothelial cells (VEC), vascular smooth muscle cells arterial (VSMCA), nociceptors (NOCI), large diameter neurons (LDN), and immune cells (IMM). **(B)** Clusters were defined using markers identified with the FindMarkers command in Seurat. The size of the dot represents the percentage of cells expressing the given gene within a cluster, and the color corresponds to the average expression (unscaled data) across all cells within a cluster for each gene of interest. **(C)** Matrisome gene expression in murine DRG bulk RNAseq data, bar chart of percentage of expressed genes for each category of the matrisome. Number inside the bar represents the mean per group. **(D)** Numbers and percentages of cells in each cluster.

**Figure S4:** Cell chat inferred cell-cell communication and pathway analysis. **(A)** Absolute number of inferred interactions in the murine DRG tissue. Arrowhead shows the direction of interaction while the thickness of the arrow is weighted based on the number of interactions. **(B)** Inferred interactions strength (weights) are represented. Arrowhead shows the direction of interaction while the thickness of the arrow is representative of the weight/strength of the interaction. The size of the dots represents the number of cells for that cell type. **(C)** Outgoing and incoming signaling role analysis on the aggregated cell-cell communication network from all found pathways. All ligand-receptor pathways are ranked based on their weight in the DRG sample. Outgoing signaling patterns show the cell type sources of the ligands of the interactions of the pathway while incoming signaling patterns indicate which cell types express the receptors of the pathway. The height of the colored bar chart on top represents the total strength of each cell type of as a source of all aggregated interactions, and the height of the right grey bar indicates the strength of the signaling pathway by summarizing all cell types. Schwann cells (SCHW), satellite glial cells (SATG), vascular leptomeningeal-/fibroblast- like cells (VLMC-like), vascular endothelial cells (VEC), vascular smooth muscle cells arterial (VSMCA), nociceptors (NOCI), large diameter neurons (LDN), and immune cells (IMM).

**Figure S5**: Violin plots of other highly contributing ligand-receptor pairs in the Collagen pathway network in identified in scRNAseq murine DRG data associated with Figure 4B. **(A)** The ligands and their expression profiles over the different clusters. **(B)** The receptors and their expression profiles over the different clusters. Schwann cells (SCHW), satellite glial cells (SATG), vascular leptomeningeal-/fibroblast- like cells (VLMC-like), vascular endothelial cells (VEC), vascular smooth muscle cells arterial (VSMCA), nociceptors (NOCI), large diameter neurons (LDN), and immune cells (IMM).

**Figure S6:** Cell chat inferred cell-cell communication and pathway analysis of the Fibronectin (Fn1) pathway. **(A)** The inferred ‘Fibronectin’ signaling network in murine DRG. Arrow points show the direction of the interaction, and the size of the arrow corresponds with the weight of interaction. Size of the dots corresponds with the relative number of cells per cell cluster. Schwann cells (SCHW), satellite glial cells (SATG), vascular leptomeningeal like cells (VLMC-like), vascular endothelial cells (VEC), vascular smooth muscle cells arterial (VSMCA), nociceptors (NOCI), large diameter neurons (LDN) and immune cells (IMM). **(B)** Each predicted ligand-receptor pair within the Fibronectin signaling network in murine DRG is ranked based on the relative contribution to the overall fibronectin signaling pathway in panel A. **(C)** Violin plots of the expression profile across the different cell types in the DRG of the contributing ligands and receptors in the fibronectin pathway network.

**Figure S7:** Cell chat inferred cell-cell communication and pathway analysis of the heparan sulfate proteoglycan (Hspg) pathway. **(A)** The inferred ‘heparan sulfate proteoglycan ’ signaling network in murine DRG. Arrow points show the direction of the interaction, and the size of the arrow corresponds with the weight of interaction. Size of the dots corresponds with the relative number of cells per cell cluster. Schwann cells (SCHW), satellite glial cells (SATG), vascular leptomeningeal like cells (VLMC-like), vascular endothelial cells (VEC), vascular smooth muscle cells arterial (VSMCA), nociceptors (NOCI), large diameter neurons (LDN) and immune cells (IMM). **(B)** Each predicted ligand-receptor pair within the heparan sulfate proteoglycan signaling network in murine DRG is ranked based on the relative contribution to the overall fibronectin signaling pathway in panel A. **(C)** Violin plots of the expression profile across the different cell types in the DRG of the contributing ligands and receptors in the heparan sulfate proteoglycan pathway network.

**Figure S8.** Cell chat inferred cell-cell communication and pathway analysis of the Semaphorin 3 (Sema3) pathway. **(A)** The inferred ‘Semaphorin 3A’ signaling network in murine DRG. Arrow points show the direction of the interaction, and the size of the arrow corresponds with the weight of interaction. Size of the dots corresponds with the relative number of cells per cell cluster. Schwann cells (SCHW), satellite glial cells (SATG), vascular leptomeningeal like cells (VLMC-like), vascular endothelial cells (VEC), vascular smooth muscle cells arterial (VSMCA), nociceptors (NOCI), large diameter neurons (LDN) and immune cells (IMM). **(B)** Each predicted ligand-receptor pair within the Semaphorin 3A signaling network in murine DRG is ranked based on the relative contribution to the overall fibronectin signaling pathway in panel A. **(C)** Violin plots of the expression profile across the different cell types in the DRG of the contributing ligands and receptors in the Semaphorin 3A pathway network.

**Figure S9:** Cell chat inferred cell-cell communication and pathway analysis of the Vascular endothelial growth factor (Vegf) pathway. **(A)** The inferred ‘Vascular endothelial growth factor’ signaling network in murine DRG. Arrow points show the direction of the interaction, and the size of the arrow corresponds with the weight of interaction. Size of the dots corresponds with the relative number of cells per cell cluster. Schwann cells (SCHW), satellite glial cells (SATG), vascular leptomeningeal like cells (VLMC-like), vascular endothelial cells (VEC), vascular smooth muscle cells arterial (VSMCA), nociceptors (NOCI), large diameter neurons (LDN) and immune cells (IMM). **(B)** Each predicted ligand-receptor pair within the Vascular endothelial growth factor signaling network in murine DRG is ranked based on the relative contribution to the overall fibronectin signaling pathway in panel A. **(C)** Violin plots of the expression profile across the different cell types in the DRG of the contributing ligands and receptors in the Vascular endothelial growth factor pathway network.

**Figure S10:** Cell chat inferred cell-cell communication and pathway analysis of the Fibroblast growth factor (Fgf) pathway. **(A)** The inferred ‘heparan sulfate proteoglycan ’ signaling network in murine DRG. Arrow points show the direction of the interaction, and the size of the arrow corresponds with the weight of interaction. Size of the dots corresponds with the relative number of cells per cell cluster. Schwann cells (SCHW), satellite glial cells (SATG), vascular leptomeningeal like cells (VLMC-like), vascular endothelial cells (VEC), vascular smooth muscle cells arterial (VSMCA), nociceptors (NOCI), large diameter neurons (LDN) and immune cells (IMM). **(B)** Each predicted ligand-receptor pair within the heparan sulfate proteoglycan signaling network in murine DRG is ranked based on the relative contribution to the overall fibronectin signaling pathway in panel A. **(C)** Violin plots of the expression profile across the different cell types in the DRG of the contributing ligands and receptors in the heparan sulfate proteoglycan pathway network.

**Figure S11**: Spatial transcriptomics on human DRG. **(A)** After scaling and normalization in Seurat, expression thresholds were established for each gene of interest by using the 25^th^ quartile cutoff (green box) of barcodes > 0 (black box) for that gene in each DRG sample*. SCN10A* and *DCN* are shown here as examples for each DRG, but this method was also applied to genes in parts B and C. **(B)** Percentage of co-expression of *COL6A1, COL6A2 and COL6A3* respectively with *SCN10A* or *DCN*. **(C)** Percentage of co-expression of *ITGA3, ITGAV* and *ITGB8* respectively with *SCN10A* or *DCN. SCN10A-DCN* double positive cells were excluded from analyses. n = 4 male (filled symbol), n = 5 female (open symbol).

**Figure S12:** Expression of *COL1A1, SCN10A* and *DCN* in Human DRG. **(A)** RNAscope used to identify cells expressing *SCN10A* and *COL1A1* or **(B)** *DCN* and *COL1A1* in human DRG (male donors n = 2). Representative sections are shown for *COL1A1* staining with the cellular marker, either with phase contrast or nuclei staining (DAPI) overlay. Scale bar = 50μm (A,B).

**Figure S13:** Negative control immunohistochemistry from human DRG incubated with species-specific secondary antibodies, but no prior exposure to rabbit anti-NeuN, DAPI, or mouse anti-GPC3. The only signal present is autofluorescence from lipofuscin. (Top row: male donor n = 1, bottom row: female donor n = 1) Scale = 25µm.


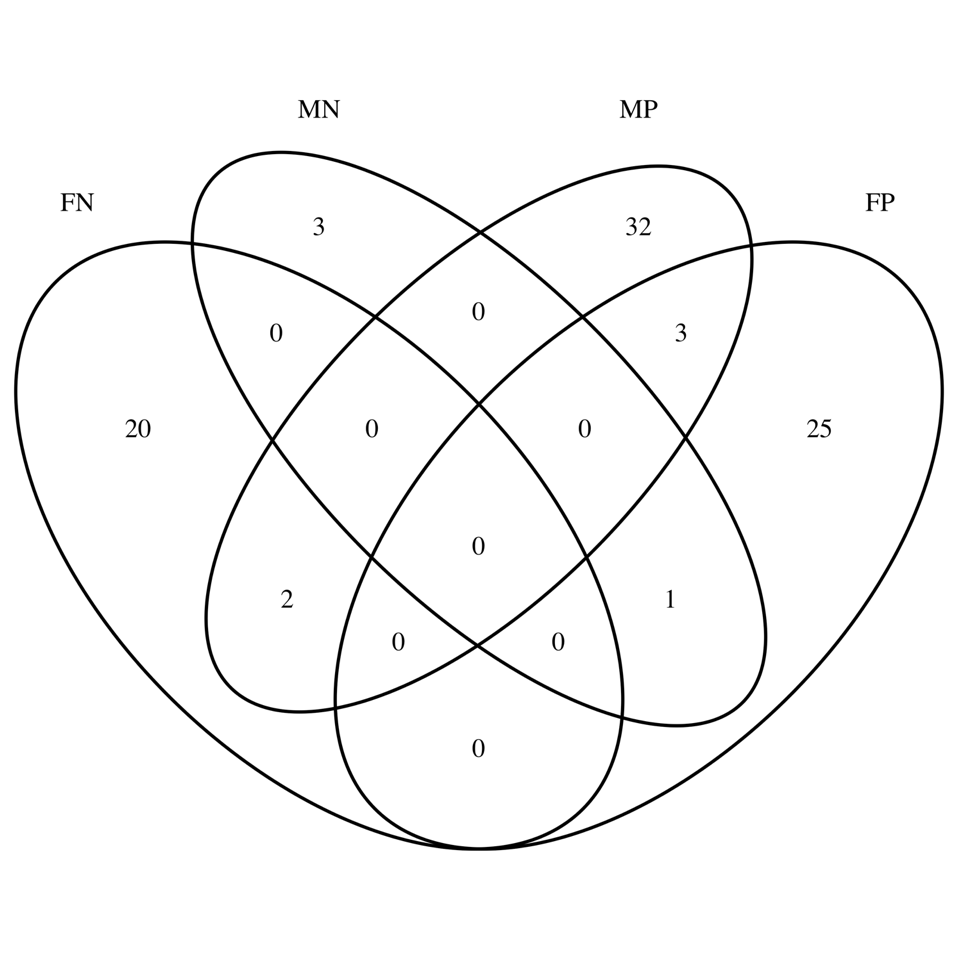


**Figure S14:** Human differentially expressed genes were selected based on the matrisome list and the human DRG bulk RNAseq data publicly available of patients with no pain or with neuropathic pain (Supplementary Table 1) (Ray et al., 2022). Venn diagram showing the overlapping DEG between different pain states per sex: FP: female pain, FN: female no pain, MP: male pain, MN: male no pain.
